# Oxidative DNA Damage and Repair: Mechanisms, Mutations, and Relation to Diseases

**DOI:** 10.3390/antiox12081623

**Published:** 2023-08-17

**Authors:** Marina Roginskaya, Yuriy Razskazovskiy

**Affiliations:** 1 Department of Chemistry, East Tennessee State University, Johnson City, TN 37614, USA; 2 Department of Physics and Astronomy, East Tennessee State University, Johnson City, TN 37614, USA; raiskazy@etsu.edu

Oxidative DNA damage (ODD) by reactive oxygen species (ROS) or reactive nitrogen species (RNS) is an inevitable tradeoff for using oxidation processes by living cells as a source of energy. A condition of oxidative stress (OS) occurs in biosystems when production and accumulation of ROS/RNS exceed the ability of biosystems to detoxify these reactive products. Such factors as ionizing radiation, UV light, tobacco smoking, pollutants, trauma, and infections can cause OS and concomitant ODD in biosystems [1,2,3].

ODD includes a multitude of DNA lesions such as nucleobase mutations, strand breaks, DNA–DNA, and DNA–protein crosslink products. To cope with DNA damage, living cells have developed elaborate DNA repair machinery. DNA repair is a complex hierarchical process that enables a single DNA oxidative lesion to modulate a multitude of pathways. Mechanisms of DNA repair remain poorly understood. In mammalian cells, ODD is repaired by two overlapping pathways: the nucleotide excision repair (NER) pathway, which removes DNA helix-distorting bulky DNA lesions, and the base excision repair (BER), which repairs small non-bulky lesions. Unrepaired ODD and failed repair attempts are directly linked to spontaneous mutagenesis, which, in its turn, is a causative factor in carcinogenesis and other debilitating diseases [1,2,3].

This Special Issue ‘Oxidative DNA Damage and Repair: Mechanisms, Mutations, and Relation to Diseases’ consists of four research articles and one review from leading experts in the field. It highlights the most recent advances in understanding mechanisms of ODD and repair, and association of these processes with cancer and other pathologies, thus making this Special Issue an essential read for anyone interested in this exciting area.

In mammals, five DNA glycosylases play a central role in maintaining genome integrity during oxidative stress by recognizing oxidized base lesions and initiating their repair via the BER pathway. The three DNA glycosylases, Nei-like1–3 (NEIL1–3), are members of the Fpg/Nei superfamily, and they recognize both purine and pyrimidine oxidation products [4]. DNA glycosylase NEIL2 plays specialized functions such as preferential repair of oxidized lesions from transcribed genes, modulation of the immune response, active DNA demethylation, and maintenance of the genome integrity [5].

Holst et al. [6] have demonstrated for the first time that NEIL2 is phosphorylated by the two kinases in vitro and in human neuroblastoma cells: cyclin-dependent kinase 5 (CDK5) and protein kinase C (PKC). Post-translational modifications such as phosphorylation are generally known to serve as molecular switches providing a fast and fine-tuned response to acute oxidative stress. Importantly, the authors demonstrated that this phosphorylation is rapidly reversed in response to oxidative stress rendered by treatment with hydrogen peroxide in human neuroblastoma cells. In paper [6], a working model of the phosphorylation state of NEIL2 is discussed. At a basal level, NEIL2 is phosphorylated by CDK5/PKC. Under oxidative stress, NEIL2 is dephosphorylated by a yet unknown phosphatase and/or by inhibition of kinase phosphorylation. Dephosphorylation lifts the inhibitory effect of phosphorylation on NEIL2 activity and promotes NEIL2-initiated DNA repair response to counteract the threat to the genome. However, the mechanism of NEIL2 dephosphorylation and possible re-phosphorylation to its basal state after oxidative stress remains to be determined [6].

Combination therapy by using more than one therapeutic agent benefits cancer treatment by reducing the drug resistance [7]. Physapruin A (PHA), a Physalis peruviana-derived withanolide (a naturally occurring steroid), is known to exhibit antiproliferation activity against oral and breast cancer cells [8]. However, its potential antitumor effects in combined treatments remain unclear. The investigation of Peng and colleagues [9] focuses on evaluating the impact of the combined treatment of ultraviolet-C with PHA (UVC/PHA) on the proliferation of oral cancer cells. The UVC/PHA combination treatment showed a greater extent of cell cycle and cell proliferation modulating, apoptosis-inducing, and DNA-damaging effects than UVC or PHA treatments of oral cancer cells alone. Importantly, the UVC/PHA treatment exhibited an improved antiproliferation effect in oral cancer cells compared to UVC or PHA alone. Thus, the authors found out that using UVC/PHA is a promising strategy for suppressing the proliferation of oral cancer cells in the absence of inhibitory effect on normal cells.

It has been known for a while that the diet containing black raspberries (BRB) reduces the level of DNA damage and carcinogenesis in cell cultures and animal models [10,11]. Sales et al. [12] have demonstrated that the inhibition of oxidatively induced DNA damage in human HeLa cells treated with black raspberry extracts (BRBE) is associated with a significant growth of the nucleotide excision repair (NER) yields of a bulky deoxyguanosine adduct derived from the polycyclic aromatic carcinogen benzo[a]pyrene (BP-dG) and a non-bulky DNA lesion, guanidinohydantoin (Gh). These effects are correlated with an increase in the expression of the critically important NER factor XPA and the helicase XPB, but not the helicase XPD. BER mechanism, unlike the NER mechanism, is not sensitive to BRBE treatment.

The apurinic/apyrimidinic (AP) site (also known as the abasic site) is a common highly mutagenic DNA lesion containing no nucleobase, which is formed either spontaneously or due to DNA damage. AP endonuclease 1/redox effector-1 (APE1/Ref-1) is the major AP endonuclease in mammalian cells. It functions via the BER pathway to create a suitable substrate for DNA polymerases [13,14]. Xue and Demple [15] investigated the properties of the Ape1-null in CH12F3 and HEK293 FT cell lines. The authors tested the Ape1 endonuclease inhibitor Compound **3** and the redox inhibitor APX2009. They demonstrated that Ape1-null cell lines were modestly more sensitive to killing by an alkylating agent than their Ape1-proficient cells. Surprisingly, the knockout cell lines showed equal sensitivity to direct killing by either inhibitor, despite the lack of the target protein.

Spinal cord injury (SCI) is the damage to the spinal cord resulting from either trauma or pathology (e.g., cancer). SCI is a severely debilitating neurological disorder which affects up to 500,000 people worldwide annually [16]. Oxidative stress plays a hallmark role in pathophysiology of SCI. Although it would be natural to assume that the consequent oxidative DNA damage is a major contributor to the pathogenesis of SCI, there is surprisingly little experimental evidence supporting this assumption, as reflected in the review by Scheijen et al. [16]. There is an agreement among researchers that oxidative DNA damage increases after SCI, primarily by using comet assays and immunohistochemistry. However, there is great variability in the timing and magnitude of the oxidative DNA damage, likely due to differences in experimental models, measurement techniques, and precision of experimental methods. For SCI, only limited evidence is present on oxidative base lesions, even for the staple oxidative base lesion 8-hydroxy-2′-deoxyguanosine (8-oxodG). While there is clear evidence that the levels of 8-oxodG increase after SCI, the timing of 8-oxodG formation and its quantities are uncertain. Similarly, the data on the amounts and timing of DNA strand breaks formation during SCI are conflicting in different research papers. There is very little data on DNA repair mechanisms in response to SCI, and only a few repair components have been assessed, such as PCNA, PARP1, and APEX1. Evidently, many gaps in knowledge of DNA damage and repair during SCI must be filled in future studies [16].

We would like to express our appreciation for the authors who have contributed to this Special Issue ‘Oxidative DNA Damage and Repair: Mechanisms, Mutations, and Relation to Diseases’ with their high-quality scientific works.
